# A cross-sectional study of knowledge and awareness of type 2 diabetes mellitus in a student population in Ghana: do demographics and lifestyle make a difference

**DOI:** 10.1080/21642850.2019.1637261

**Published:** 2019-07-04

**Authors:** Margaret Amankwah-Poku

**Affiliations:** Department of Psychology, University of Ghana, Accra, Ghana

**Keywords:** Diabetes, knowledge and awareness, student, Ghana, demographics, diabetes education

## Abstract

**Objective:**

To determine the level of knowledge and awareness of type 2 diabetes among undergraduate students and to investigate demographic and lifestyle variables associated with students’ level of knowledge and awareness.

**Design:**

Students from the University of Ghana (*n* = 726) were administered questionnaires to assess their knowledge and awareness in specific aspects of type 2 diabetes, namely, symptoms, treatment and complications of the illness.

**Main Outcome Measures:**

Level of type 2 diabetes knowledge and awareness.

**Results:**

Knowledge and awareness were higher for diabetes treatment (than for diabetes symptoms and complications), with females having more knowledge and awareness than males. Significant differences were also found in the level of knowledge and awareness of students based on their discipline of study but not the number of years of study in the university. Also, students who engaged in physical exercise showed a higher level of general knowledge and awareness of type 2 diabetes. Finally, a family history of diabetes resulted in more knowledge and awareness of type 2 diabetes.

**Conclusions:**

Education in type 2 diabetes is needed to make individuals more aware of the illness and take preventive measures. The fact that participants’ discipline of study produced differences in diabetes knowledge and awareness, suggests how education can make a difference in creating awareness.

## Introduction

### Background/rationale

Diabetes mellitus is a group of metabolic diseases characterised by chronic high blood glucose levels, due to deficiency in insulin secretion, insulin action or both (World Health Organisation [WHO], 2016). The three main types of diabetes are; type 1 diabetes, type 2 diabetes and gestational diabetes, and the less common types of diabetes are monogenic diabetes and secondary diabetes (IDF, 2017). According to the International Diabetes Federation (IDF), in 2017, p. 425 million people had diabetes and this figure was estimated to reach 629 million in 2045 (IDF, 2017). Most (90%) individuals with diabetes have type 2 diabetes. The causes of type 2 diabetes are not completely understood but are widely believed to result from a combination of genetic and lifestyle factors (IDF, 2017).

Type 2 diabetes is on the increase as a result of obesity, aging, physical inactivity, history of gestational diabetes, impaired glucose tolerance, family history of diabetes and race/ethnicity (CDC, 2011). Thus, modifying factors such as obesity, poor diet and nutrition, physical inactivity, smoking, among others, can reduce the risk of type 2 diabetes (IDF, 2017). Blacks, specifically African Americans, are for instance 1.7 times more likely to develop type 2 diabetes than Whites are, and they are more likely to experience more disabilities from complications of diabetes (Centers for Diseases and Prevention [CDC], 2011). In fact, the death rate for blacks with diabetes is 25% higher than the death rate for whites (CDC, 2011). Over the past 30 years, prevalence of diabetes among blacks have quadrupled (IDF, 2013).

The prevalence rate of diabetes in Ghana in 2014, was 3.3% with 450,000 people (20–79 years) with diabetes and 337,900 adult cases undiagnosed (IDF, 2017). In 2017, 518,400 Ghanaians had diabetes with 257,600 adult cases undiagnosed and a prevalence rate estimated at 3.6%. The increase in prevalence (in 2017) by 0.3% is evident in increasing cases of diabetes in Ghana, as well as the cost of treatment. Treatment of diabetes in Ghana in 2014 cost $148.4, per person and diabetes-related deaths recorded in that year was 8528 (IDF, 2017). This cost of treatment decreased in 2017 to $106.5 although deaths recorded increased to 9778 (IDF, 2017).

The fact that one person dies from diabetes every six seconds (IDF, 2013) makes this chronic illness alarming. Unfortunately, several hundreds of adults go undiagnosed with diabetes. Knowledge of type 2 diabetes in the general population can lead to early diagnosis when individuals recognise any symptoms, delay the onset of diabetes complications and possibly reduce diabetes-related deaths. Having knowledge and awareness about the illness is also important for behaviour change. For instance, knowledge about the risk factors of type 2 diabetes could increase awareness about the illness for individuals to modify these risk factors and change their lifestyles, thereby reducing or delaying the chances of being diagnosed with the illness.

Relative levels of knowledge and awareness of diabetes have been reported among several healthy populations, although these knowledge levels may not be adequate to prevent diagnosis of diabetes (e.g. Abdoli & Tavana, 2013; Al Shafaee et al., 2008; Alanazi et al., 2017; Deepa et al., 2014; Fezeu, Fointama, Ngufor, Mbeh, & Mbanya, 2010). Ding, Teng, and Koh (2006), for example, have reported that although the knowledge level of healthy individuals in Malaysia was above average (64%), individuals were less aware of the risk factors of diabetes, such as aging and pregnancy. Mohammed, Al-Aaragi, and Merzah (2018), have also reported overall scores regarding knowledge, attitude, and practice among a student sample (in Iraq) to be good and acceptable but recommended better educational programme to improve awareness. Again, in spite of the good general knowledge of diabetes mellitus reported among a Nigerian sample of civil servants, education on the signs and symptoms of diabetes was recommended (Agu, Agu, Nnaji, & Ugochukwu, 2014).

Poor knowledge in several aspects of diabetes (causes, complications, management and prevention) have also been recorded among Saudi nationals and recommendations made for increased awareness of the illness (Alanazi et al., 2017). A systematic review confirmed this need to increase knowledge and awareness about diabetes among the Saudi nationals (Alanazi et al., 2018). In like manner, the low knowledge of diabetes risk factors, management, and care in a Pakistani general population (41% being students) has suggested the need for public education in diabetes prevention and treatment (Gillani et al., 2018). In Oman, adult residents lacked the knowledge that was required to prevent and reduce diagnosis with diabetes (Al Shafaee et al., 2008).

Demographic variables such as level of education, age, sex and family history of type 2 diabetes have been associated with knowledge about risk factors, symptoms, treatment and complications of diabetes. Among the Oman sample, their knowledge about diabetes was directly influenced by their level of education and family history of diabetes, which perhaps increased their awareness (Al Shafaee et al., 2008). Also, compared with females, males reported more awareness of risk factors of diabetes, as well as its treatment and complications (Deepa et al., 2014; Fezeu et al., 2010; Gillani et al., 2018). Again, Jordanian students with a family history of diabetes had better knowledge about the illness. The fourth year Jordanian students were more educated about diabetes than the first year students, suggesting the positive impact of higher education on knowledge about diabetes (Al-Sarayra & Khalidi, 2012). These findings and many more (e.g. Abdoli & Tavana, 2013; Maina, Ndegwa, Njenga, & Muchemi, 2010; Nisar, Khan, Qadri, & Sher, 2008; Soltanian, Bahreini, & Afkhami-Ardekani, 2007), suggest the need for intensive diabetes education to increase the knowledge level among healthy individuals.

Studies conducted in Ghana on diabetes are minimal and have not focused on knowledge and awareness about diabetes. While some studies have investigated the prevalence of diabetes (e.g. Abubakaria & Bhopal, 2007; Gatimu, Milimo & Sebastian, 2016), others have investigated predictors of glycemic control (Apini, Annan, Apprey, & Asamoah-Boakye, 2018) and factors associated with diabetes (e.g. Danquah et al., 2012). Kugbey, Kugbey, Asante, and Adulai (2017) studied knowledge of diabetes but this was in relation to patients’ self-care practices. All of these studies have tested patients and not a general population. Thus, to the best of the author’s knowledge, this study is the first to investigate knowledge and awareness about diabetes in Ghana in a student population.

In recent times, the adaptation of western lifestyles, coupled with busy lives and sedentary lifestyles have resulted in Ghanaians turning more and more to fast foods without perhaps knowing the implications for health. Knowledge of type 2 diabetes may be the bases for lifestyle changes in order to prevent diagnosis with the illness. This makes it necessary to assess the knowledge and awareness levels of individuals and educate them about the illness in order to detect early symptoms and seek medical attention.

The fact that diabetes is on the increase makes it necessary to use preventive means, such as education for lifestyle changes, to reduce its prevalence in Ghana. Type 2 diabetes is most common in older adults, however, over time, due to increasing levels of obesity, poor diet and physical inactivity, this condition is now diagnosed among children, adolescent and younger adults (IDF, 2017). This makes university students as susceptible to developing this illness as older adults may be. Being in university is often associated with the independence and freedom to make food choices and engage in lifestyles that may not promote healthy living. For instance, weight gain has been reported among university/ college students (Fedewa, Das, Evans, & Dishman, 2014; Gropper, Simmons, Connell, & & Ulrich, 2012). Plotnikoff et al. (2015) in a systematic review of health behaviours (physical activity, weight loss and diet interventions), recommended universities/ colleges as ideal settings for implementing programmes that promote health since a large number of young adults can be targeted (Plotnikoff et al., 2015). Thus, studying university students are targeting a population that may be susceptible to a lifestyle conditions such as type 2 diabetes and (based on the findings), providing the needed education for the prevention or early diagnoses of type 2 diabetes.

As mentioned above, demographic variable has been associated with individual’s level of knowledge in diabetes (e.g. Al Shafaee et al., 2008; Al-Sarayra & Khalidi, 2012; Deepa et al., 2014; Fezeu et al., 2010; Gillani et al., 2018). Thus, it will not be sufficient just to know the level of knowledge and awareness of type 2 diabetes but also to know what factors may determine a person’s level of knowledge. This way, one would know what variables are worth targeting (such as gender, degree of study, eating habits, etc.), for effective diabetes education among students.

### Objectives

This study, therefore, aimed to (a) determine the level of knowledge and awareness of type 2 diabetes among undergraduate students, (b) investigate demographic variables (age, sex, year of study and discipline of study) associated with students’ level of knowledge and awareness of type 2 diabetes and (c) investigate lifestyle variables (physical activity and healthy eating) association with students’ level of knowledge and awareness of type 2 diabetes. This will provide insight into the knowledge and awareness of type 2 diabetes in a student population and determine the areas of knowledge deficit. Consequently, it will help to determine the need for diabetes education.

## Methods

### Study design

A cross-sectional study was conducted among university undergraduate students to assess their knowledge and awareness of the symptoms, treatment and complications of diabetes and the role of demographic and lifestyle variables.

### Setting

This study was conducted at the University of Ghana, Legon. The university is located in Accra, which is the capital of Ghana and the capital of the Greater Accra Region. The University of Ghana academic structure is made of four colleges with the colleges made up of schools and the schools made up of departments. Questionnaire was administered in lecture rooms and data were collected from September 2015 to November 2015.

### Participants

A total of 726 participants were included in this study. Participants were University of Ghana undergraduate students who were recruited from three disciplines, namely, Family and Consumer Sciences, Business Administration and Psychology. The fishbowl technique was used to select the three disciplines from which participants were recruited. First, the names of the four colleges (Health Sciences, Basic and Applied Sciences, Humanities and Education) were written on pieces of papers, folded and put in a bowl. A volunteer unrelated to the study was then made to pick two out of the four colleges. The College of Basic and Applied Sciences and Humanities were randomly selected. Since the latter is a larger college with 16 schools compared to the former with 10 schools, one school was selected from the College of Basic and Applied Sciences, while two schools were selected from the College of Humanities. The fishbowl technique was further used to randomly select the schools, and then finally the departments, which yielded a random selection of Family and Consumer Sciences, Business Administration and Psychology. For each discipline, students were recruited from each of the four-year levels of study (levels 100, 200, 300 and 400).

### Variables

The independent variables investigated were (a) demographic variable which included age, sex, year of study and discipline of study and (b) lifestyle variable which included assessing healthy eating and physical exercise. The outcome variables measured were general knowledge and awareness of type 2 diabetes, diabetes symptoms, diabetes treatment and diabetes complications. Students diagnosed with diabetes were excluded as their knowledge in diabetes could confound the outcome of the study.

### Data source/measurement

*Demographic and lifestyle information –* Demographic information obtained, included, age, sex, weight, height, year of study, discipline of study, as well as lifestyle questions determining whether or not participants were maintaining healthy eating or were exercising.

*Development of Knowledge and Awareness of Diabetes Scale* (*KADS*) *–* A Diabetes Knowledge and Awareness Scale (50 items) was developed by the researcher after intensive literature review and review of existing measures on knowledge and awareness of diabetes that have been used by other researchers (e.g. Fezeu et al., 2010; Wee, Ho, & Li, 2002). Items on the scale assessed knowledge in type 2 diabetes. The scale had three subscales measuring symptoms of diabetes (15 items), treatment of diabetes (19 items) and complications of diabetes (16 items). Items on all three subscales included, true and false statements in order to avoid participants giving set responses or obtaining high scores by chance. Scoring of responses were ‘True’ (=1), ‘False’ (=0) and ‘Don’t Know’ (=0). Thus, a correct response was scored as ‘1’ mark, while a wrong response or ‘don’t know’ was scored as ‘0’, and total scores were obtained by adding up correct responses. Higher scores on the subscales indicated higher levels of knowledge and awareness in a specific aspect of diabetes, while lower scores indicated lower levels of knowledge and awareness.

The 50-item scale was pilot tested for comprehensibility in a sample of 50 students from the three disciplines at the University of Ghana, namely, Psychology (*n* = 20), Business Administration (*n* = 15), and Family and Consumer Sciences (*n* = 15) with a mean (SD) age of 20.7(1.8) years. Based on feedback received, and reliability tests performed, a final questionnaire of 44 items made up of 11 items for symptoms of diabetes, 17 items for treatment of diabetes and 16 items for complications of diabetes was devised with the above-mentioned responses. Cronbach’s alpha obtained were as follows; symptoms of diabetes, *α* = .81, treatment of diabetes *α *= .73 and complications of diabetes *α *= .76. Cronbach’s alpha for the actual data collected among the 726 participants were, symptoms of diabetes, *α *= .75, treatment of diabetes *α *= .72 complications of diabetes *α* = .77. These three together formed student’s general knowledge and awareness of diabetes (*α* = .88).

### Bias

Biases have the potential to confound research findings and therefore multiple steps were taken to address this. First, the cross-sectional design was used, engaging students in Core courses (i.e. compulsory courses) to ensure maximum enrolment in the study. This way, the researcher was certain that all students in the various disciplines were available and had equal chance of participating. Second, in terms of selecting the population, the fishbowl technique was used to select the disciplines of study. Thus, every discipline had an equal chance to be selected. Third, to avoid selection bias during recruitment, students were excluded if they had been diagnosed with diabetes and if they did not belong to any of the three disciplines studied.

### Study size

Power analysis was conducted, using the statistical tool G*Power (Faul, Erdfelder, Lang, & Buchner, 2007), to determine the minimum sample size for the study. Following Cohen and Cohen’s (1983) guidelines for power analysis, an alpha level of .05, a statistical power of .80, and a medium effect size, a minimum sample size of 270 was estimated to be adequate for the purpose of this study. However, this number was increased to allow for the testing of a much larger sample. Thus, a total number of 803 students were conveniently sampled and questionnaires were administered to them. Out of this number, 726 participants completed the questionnaires.

### Quantitative variables

As indicated above, variables assessed were demographic variables, lifestyle variables and the level of knowledge and awareness in diabetes. Responses of participants were coded in the Statistical Package for Social Sciences (SPSS) version 20 software, for analyses. Corrects responses to the knowledge and awareness in diabetes questions were scored as one (1) mark and total scores were determined.

### Procedure

Participants were recruited from lecture halls during lectures. The researcher first contacted lecturers in the various disciplines and sought their permission to administer the questionnaires. In the lecture halls, the researcher explained the purpose of the study to the prospective participants and asked for voluntary participation. The researcher together with research assistants then distributed the questionnaires to those who consented to participate, to complete. Questionnaires were distributed just before a lecture begun or at the end of a lecture for participants to complete and submit before leaving the lecture hall. This had an added advantage as the researcher and research assistants were at hand to ensure all responses were completed. It must be noted however that some of the participants withdrew from the study and therefore choice not to complete their questionnaires. Data was collected over a period of four weeks.

### Statistical methods

Descriptive and inferential statistics were performed using the IBM SPSS. The independent variables studied were sex, year of study, discipline of study, healthy eating and exercising, while the outcome variables were related variables (general knowledge and awareness of type 2 diabetes, diabetes symptoms, diabetes treatment and diabetes complications). Also, data obtained conformed to the assumptions of MANOVA; thus, a factorial MANOVA and a One-way MANOVA were used to compare means to test the influence of demographic and lifestyle variables on knowledge and awareness of diabetes. Significant levels for all the results were 0.05, 0.01 and 0.001. Where the results showed significant differences, post-hoc analysis with Bonferroni correction was used. No missing data was recorded for the 726 participants included in the study. This was possible because when participants handed in their completed questionnaires, the researcher and research assistants check the questionnaires to ensure all items had been responded to.

### Ethics Statement

Ethical approval to conduct this study was obtained from the Ethics Committee for Humanities at the University of Ghana. Participants gave written informed consent to participate in the study.

## Results

### Participant

Out of the 803 participants recruited, 726 participants completed the questionnaires. The rest of the 77 participants did not fully complete their questionnaires so their data were excluded. Thus, the non-response rate in this study was 9.6%. These 77 participants withdrew from the study because they were no longer interested due to the lengthy nature of the questionnaire and/ were in a hurry to leave the lecture hall for their next lecture or for other appointments.

### Descriptive data

Descriptive statistics are presented to show the level of knowledge and awareness of diabetes reported by participants.

#### Participants’ demographic and lifestyle characteristics

Demographic characteristics of participants are presented in Table 1. The mean (SD) age of participants was 20.6(1.9) years with more than half of them being females, (54.5%). There were relatively equal number of students from the three disciplines, namely Psychology (34.4%), Family and Consumer Science (32.9%) and Business Administration (32.8%). There were more level 300 (31.5%) and level 200 (30.3%) students than level 400 (20.2%) and level 100 (17.9%) students. About one-third of the participants (31.1%) indicated that they had family members with diabetes, most of whom were extended family members (66.3%). While the majority of participants reported that they maintained healthy eating (75.9%), less than half of them reported that they engaged in physical exercise (43.8%). Of the various sources of information on diabetes, many participants indicated newspapers and magazines as their source of diabetes information, (85.8%), followed by information from schools (63.1%) and then from lectures and seminars (55.1%). The least reported source of information was from family and friends (19.6%) followed by posters (21.6%). Self-reported weight and height were obtained from participants; however, 38% could not provide their weight, while 57.5% could not provide their height. As a result, accurate body mass index (BMI) could not be obtained, hence, weight and height measures were excluded from the analysis.

**Table 1.** Demographic characteristics of participants (*n* = 726).

Variable	*n* (%)
*Age –* Mean(SD) 20.6(1.9)	
Sex	
Male	330 (45.5)
Female	396 (54.5)
Degree of study	
Bsc family and consumer sciences	239 (32.9)
Bsc business administration	238 (32.8)
Psychology	249 (34.3)
Level of study	
Level 100	130 (17.9)
Level 200	220 (30.3)
Level 300	229 (31.5)
Level 400	147 (20.2)
*Family history of diabetes*	
Yes	226 (31.1)
No	500 (68.9)
*Type of family members with diabetes*	
Nuclear family	57 (25.2)
Extended family	152 (67.3)
Both	14 (6.2)
Did not indicate	3 (1.3)
*Maintaining healthy eating*	
Yes	551 (75.9)
No	169 (23.3)
Did not indicate	6 (0.8)
*Engaging in physical exercise*	
Yes	318 (43.8)
No	401 (55.2)
Did not indicate	7 (1.0)
*Sources of information*	
Newspaper and magazine	623 (85.8)
School	458 (63.1)
Lectures and seminars	400 (55.1)
Social function	357 (49.2)
Television and radio	289 (39.8)
Health facility	278 (38.3)
Church, Mosque	188 (25.9)
Posters	157 (21.6)
Family and friends	142 (19.6)
Other sources	31 (4.3)

### Outcome data

#### Participants’ responses to specific measures of type 2 diabetes knowledge and awareness

From Table 2, overall, knowledge and awareness was higher for diabetes treatment (64.8%) than for complications (44.6%) and symptoms (41.2%). Of the 11 questions asked about diabetes symptoms, there were only 4 questions (3, 4, 7 and 8) in which more than half of the participants responded correctly. For diabetes treatment, of the 17 questions, many participants responded correctly to most of the questions except 5 items (3, 8, 12, 15, 17) in which less than half of the participants responded incorrectly. For instance, for question 15, just about 10% of the participants responded correctly. Finally, for diabetes complications, more than half of the participants made correct responses to 9 questions while less than half of them were able to respond correctly to the rest (1, 3, 5, 8, 9, 13, 16). Less than 10% of the participants responded correctly to questions 5 and 8. Thus, overall, on all the questions, a little over half of the participants (51.5%) had some general knowledge and awareness of type 2 diabetes.

**Table 2.** Correct responses of participants on knowledge and awareness of type 2 diabetes scale.

Questions	*n*	%
**General knowledge and awareness**	***726***	***51****.****5***
**Symptoms of diabetes**	***726***	***41****.****2***
1. Excessive thirst is a symptom of diabetes	178	24.5
2. Weight loss is a symptom of diabetes	215	29.6
3. Feeling constantly tired than usual is a symptom of diabetes	381	52.5
4. Frequent urination, especially at night, is a symptom of diabetes	391	53.9
5. Losing weight despite a normal appetite is a symptom of diabetes	225	31.0
6. Diarrhoea is a symptom of diabetes	290	39.9
7. Weakness is a symptom of diabetes	383	52.8
8. Slow healing of cuts and wounds are symptoms of diabetes	501	69.0
9. Blurriness of vision is a symptom of diabetes	251	34.6
10. Numbness of hands and feet is a symptom of diabetes	281	38.7
11. Loss of appetite is a symptom of diabetes	190	26.2
**Treatment of diabetes**	***726***	***64****.****8***
1. Regular exercise is important for diabetes treatment	627	86.4
2. Tablets are available for the control of diabetes	581	80
3. A regular exercise regimen will help to cure diabetes	179	24.7
4. People with diabetes should eat healthily	697	96.0
5. Once diabetes is controlled, exercise is no longer required	672	92.6
6. Insulin injections are available for the control of diabetes	568	78.2
7. Diabetes treatment requires good weight control	556	76.6
8. Insulin injections can cure diabetes	311	42.8
9. Once diabetes is controlled, eating restrictions are no longer required	648	89.3
10. People with diabetes can lead a normal life once their sugar levels are well controlled	588	81.0
11. No diet control is needed after tablets are taken	625	86.1
12. People with diabetes should care for their toes and feet well	315	43.4
13. People with diabetes should have low fat and high fibre diet	475	65.4
14. People with diabetes should not skip meals, especially when busy	408	56.2
15. People with diabetes should carry sweets when they go out	74	10.2
16.People with diabetes should not test their blood glucose level at home	463	63.8
17. Every person with diabetes will eventually use insulin injections	209	28.8
**Complications of diabetes**	***726***	***44***.***6***
1. When diabetes is left untreated, it can lead to blindness	239	32.9
2. Wounds in people with diabetes heal more quickly	593	81.7
3. Complication of diabetes may be seen in nerves	200	27.5
4. Poorly controlled diabetes can result in stroke	445	61.3
5. When diabetes is left untreated, it can cause back pains	37	5.1
6. Diabetes complications can cause hypertension	441	60.7
7. Diabetes complications can cause heart disease	435	59.9
8. Diabetes complications can cause cancer	69	9.5
9. Sexual impotence is not a complication of diabetes	192	26.4
10. Eye problems can result from diabetes complication	363	50.0
11. Diabetes complications can result in amputation	455	62.7
12. Diabetes complications can cause foot problems	385	53.0
13. Long term complications of diabetes cannot be prevented	221	30.4
14. Diabetes complications can cause kidney problems	393	54.1
15. Diabetes complications cannot affect a pregnant woman	378	52.1
16. Diabetes can cause male sexual dysfunction	337	46.4

#### Participants’ responses to knowledge and awareness of type 2 diabetes scale per discipline of study

In Table 3, for diabetes symptoms, psychology students made correct responses (37.1%) compared to Family and Consumer Science (34.4%) and Business Administration students (28.5%). For diabetes treatment, Family and Consumer Science students made slightly more correct responses (35.1%) than Psychology students (34.3%) and Business Administration students (30.6%). Family and Consumer Science students also made more correct responses about diabetes complications (37.1%) than Psychology (35.3%) and Business Administration (27.6%) students.

**Table 3.** Correct responses of participants on knowledge and awareness of type 2 diabetes per discipline of study (%).

Questions	Fam. Cons Sci	Admin	Psych
**Symptoms of diabetes**	***34***.***4***	***28***.***5***	***37***.***1***
1. Excessive thirst is a symptom of diabetes	33.7	31.5	34.8
3. Weight loss is a symptom of diabetes	32.6	27.4	40.0
4. Feeling constantly tired than usual is a symptom of diabetes	35.2	27.6	37.3
6. Frequent urination, especially at night, is a symptom of diabetes	33.8	28.9	37.3
7. Losing weight despite a normal appetite is a symptom of diabetes	34.7	28.9	36.4
8. Diarrhoea is a symptom of diabetes	34.5	24.1	41.4
10. Weakness is a symptom of diabetes	34.5	29.2	36.3
12. Slow healing of cuts and wounds are symptoms of diabetes	33.9	29.9	36.1
13. Blurriness of vision is a symptom of diabetes	35.9	27.9	36.3
14. Numbness of hands and feet is a symptom of diabetes	33.8	28.1	38.1
15. Loss of appetite is a symptom of diabetes	35.3	30.5	34.2
**Treatment of diabetes**	***35***.***1***	***30***.***6***	***34***.***3***
1. Regular exercise is important for diabetes treatment	33.0	32.1	34.9
2. Tablets are available for the control of diabetes	32.4	32.5	35.1
3. A regular exercise regimen will help cure diabetes	35.8	25.1	39.1
4. People with diabetes should eat healthily	33.4	32	34.6
5. Once diabetes is controlled, exercise is no longer required	33.9	31.4	34.7
8. Insulin injections are available for the control of diabetes	35.7	30.8	33.5
9. Diabetes treatment requires good weight control	33.3	31.8	34.9
10. Insulin injections can cure diabetes	36.7	28.9	34.4
11. Once diabetes is controlled, eating restrictions are no longer required	34.7	30.6	34.7
12. People with diabetes can lead a ‘normal’ life once their sugar levels are well controlled	33.0	31.3	35.7
13. No diet control is needed after tablets are taken	34.2	29.9	35.8
15. People with diabetes should care for their toes and feet well	35.6	29.8	34.6
16. People with diabetes should have low fat and high fibre diet	30.5	31.4	38.1
17. People with diabetes should not skip meals especially when busy	35.8	31.6	32.6
18. People with diabetes should carry sweets when they go out	27.0	39.2	33.8
19. People with diabetes should not test their blood glucose level at home	35.2	29.4	35.4
20. Every person with diabetes will eventually use insulin injections	42.6	23.0	34.4
**Complications of diabetes**	***37***.***1***	***27***.***6***	***35***.***3***
1. When diabetes is left untreated, it can lead to blindness	33.9	31.4	34.7
2. Wounds in people with diabetes heal more quickly	36.4	28.3	35.2
3. Complication of diabetes may be seen in nerves	41.0	27.5	31.5
4. Poorly controlled diabetes can result in stroke	34.8	29.9	35.3
5. When diabetes is left untreated, it can cause back pains	48.6	18.9	32.4
6. Diabetes complications can cause hypertension	34.9	30.2	34.9
7. Diabetes complications can cause heart disease	34.3	29.7	36.1
8. Diabetes complications can cause cancer	39.1	23.2	37.7
9. Sexual impotence is not a complication of diabetes	37.0	26.0	37.0
10. Eye problems can result from diabetes complications	34.4	30.3	35.3
11. Diabetes complications can result in amputation	33.8	29.5	36.7
12. Diabetes complications can cause foot problems	34.3	27.8	37.9
13. Long term complications of diabetes cannot be prevented	40.7	27.1	32.1
14. Diabetes complications can cause kidney problems	37.9	29.0	33.1
15. Diabetes complications cannot affect a pregnant woman	37.8	27.8	34.4
16. Diabetes can cause male sexual dysfunction	34.7	24.3	40.9

### Main results

#### Demographic and lifestyle variables influencing knowledge and awareness of type 2 diabetes

Factorial MANOVA was performed to determine whether demographic and lifestyle variables influenced participants’ knowledge and awareness of type 2 diabetes. Students’ general as well as specific knowledge and awareness of type 2 diabetes were compared based on the various demographic and lifestyle variables. Significant differences were found for gender, degree of study, family history, maintaining healthy eating and engaging in physical exercise, but not for level of study (see Table 4). However, there were no interaction effects amongst these demographic variables and the lifestyle variables for general as well as specific knowledge and awareness of type 2 diabetes. Thus, interactions effects were not reported.

**Table 4.** Factorial MANOVA comparing type 2 diabetes knowledge and awareness on demographic and lifestyle variables.

Variable	Symptoms	Treatment	Complication	Overall knowledge
**Maximum score**	**11**	**17**	**16**	**44**
Age – Mean (SD) 20.6 (1.9)	** **	** **	** **	** **
*Sex(p value)*	***F(1, 726) = 0.80, p = .37, η^2^ = .001***	F(1, 726) = 12.19, p = .001, η^2^ = .017*	***F(1, 726) = 0.78, p = .38, η^2^ = .001***	***F(1, 726) = 1.61, p = .21, η^2^ = .002***
Male	4.45 (2.85)	10.54 (3.04)	7.35 (3.65)	22.34 (8.04)
Female	4.49 (2.83)	11.41 (2.77)	6.96 (3.28)	22.96 (7.37)
*Degree of study(p value)*	F(2, 726) = 7.50, p = .001, η^2^ = .021*	F(2, 726) = 9.58, p = .0001, η^2^ = .027**	F(2, 726) = 12.06, p = 0001, η^2^ = .033**	F(2, 726) = 13.71, p = .0001, η^2^ = .038**
Bsc family and consumer	4.72 (2.78)	11.42 (2.62)	7.79 (3.36)	23.94 (7.25)
Bsc business Admin	3.94 (2.9)	10.36 (3.40)	6.21 (3.57)	20.50 (8.34)
BA psychology	4.90 (2.84)	11.25 (2.60)	7.40 (3.25)	22.68 (7.68)
*Level of study(p value)*	***F(3, 726) = 0.02, p = 1.0, η^2^ = .000***	***F(3, 726) = 1.17, p = .32, η^2^ = .005***	***F(3, 726) = 1.56, p = .20, η^2^ = .007***	***F(3, 726) = 0.81, p = .49, η^2^ = .003***
Level 100	4.70 (2.85)	10.91 (2.80)	6.75 (3.28)	22.35 (7.53)
Level 200	4.52 (2.92)	10.94 (2.82)	7.04 (3.56)	22.50 (7.82)
Level 300	4.51 (2.62)	11.09 (2.72)	7.48 (3.22)	23.07 (7.04)
Level 400	4.41 (2.80)	11.10 (3.47)	7.14 (3.46)	22.63 (8.56)
*Family history of diabetes*	*F*(1, 726) = 11.18, p = .001, η^2^ = .016*	*F*(1, 726) = 17.38, p = .0001, η^2^ = .024**	F(1, 726) = 5.26, p = .02, η^2^ = .007*	F(1, 726) = 15.15, p = .0001, η^2^ = .021**
Yes	5.25 (2.80)	11.86 (2.71)	7.93 (3.41)	25.04 (7.19)
No	4.18 (2.80)	10.59 (2.93)	6.79 (3.43)	21.55 (7.67)
*Maintaining healthy eating*	***F(1, 726) = 2.02, p = .16, η^2^ = .003***	***F(1, 726) = 0.04, p= .85, η^2^ = .000***	F(1, 726) = 12.75, p = .0001, η^2^ = .018**	F(1, 726) = 4.93, p = .03, η^2^ = .007*
Yes	4.64 (2.89)	11.03 (2.92)	7.41 (3.47)	23.09 (7.77)
No	4.10 (2.65)	10.80 (2.91)	6.25 (3.30)	21.15 (7.25)
*Engaging in physical exercise*	***F(1, 726) = 3.35, p= .07, η^2^ = .005***	***F(1, 726) = 3.38, p= .07, η^2^ = .005***	***F(1, 726) = 1.89, p= .17, η^2^ = .003***	F(1, 726) = 4.06, p = .04, η^2^ = .006*
Yes	4.83 (2.82)	11.32 (2.73)	7.68 (3.43)	23.83 (7.44)
No	4.27 (2.85)	10.71 (3.04)	6.71 (3.43)	21.69 (7.76)

*p < 0.05.

**p < 0.001.

*Gender –* There was gender difference in the knowledge and awareness of diabetes treatment as females were more knowledgeable than males (*p = .001*). However, there was no gender difference in students’ general and specific knowledge and awareness of diabetes and diabetes symptoms and complications.

*Degree of study –* Significant differences were found in the level of knowledge and awareness of students based on their discipline of study for the general and specific measures of diabetes as follows; general knowledge and awareness of diabetes (*p *= *.0001*), diabetes symptoms, (*p = .001*), diabetes treatment, (*p = .0001*) and diabetes complication, (*p = .0001*). Multiple comparison with Bonferroni correction showed that the differences were between students studying Family and Consumer Science and those studying Business Administration and also between students studying Psychology and those studying Business Administration. Family and Consumer Science students had more general knowledge and awareness of diabetes than Business Administration students, (*p = .0001*), and also more knowledge in diabetes symptoms (*p = .007*), diabetes treatment (*p = .0001*), and diabetes complications, (*p = .0001*). On the other hand, Psychology students had more general knowledge and awareness of diabetes than Business Administration students, (*p = .0001*), and more knowledge of diabetes symptoms (*p = .0001*), diabetes treatment (*p < .002*), and diabetes complications, (*p = .0001*). No significant difference was found between the knowledge and awareness level of Family and Consumer Science students and Psychology students.

*Level of study –* Students’ level of study had no significant effect on their knowledge and awareness of diabetes. This indicated that the number of years they had spent in the university did not influence their knowledge and awareness level.

*Family History –* Participants with family history of diabetes had more knowledge and awareness of diabetes than those with no family history of diabetes. The former had more knowledge and awareness of diabetes symptoms (*p = .001*)*,* diabetes treatment (*p = .0001*), diabetes complications, *p < .02* and general knowledge of diabetes (*p = .0001*).

*Maintaining healthy eating –* Knowledge and awareness level of participants who reported maintaining healthy eating was compared with those who did not maintain healthy eating, and a significant difference showed for general knowledge in diabetes (*p = .03*). This difference, however, was only evident for diabetes complications (*p = .0001*), with students who maintained healthy eating, having higher level of knowledge and awareness of diabetes.

*Physical exercise –* Students who reported that they engaged in physical exercise had more general knowledge and awareness of diabetes than those who did not engage in physical exercise (*p = .04*). Differences in diabetes complications were not significant, (*p = .17*), and that for diabetes symptoms and treatment only approached borderline significance (*p = .07*).

#### Knowledge and awareness of type 2 diabetes – comparing levels of study per discipline of study

Knowledge and awareness level amongst the levels of study (levels 100, 200, 300 and 400) were compared within each of the three disciplines using a One-way MANOVA. With the exception of one comparison, no comparisons within the disciplines of study yielded significant results.

*Bsc. Family and Consumer Science –* Level of knowledge and awareness of participant did not differ per their levels of study (levels 100, 200, 300 and 400). Results for the general knowledge and awareness of diabetes was *F(3, 726) = 0.05, p = .99, η^2 ^= .001* and for the specific measures of diabetes as follows; diabetes symptoms, *F(3, 726) = 0.74, p = .53, η^2 ^= .009*, diabetes treatment, *F(3, 726) = 0.22, p = .88, η^2 ^= .003* and diabetes complication, *F(3, 726) = 1.10, p = .35, η^2 ^= .014*.

*BA. Psychology –* Results comparing the 4 levels of study were as follows; the general knowledge and awareness of diabetes, *F(3, 726) = 0.52, p = .67, η^2 ^= .006*, and specific measures of diabetes as follows; diabetes symptoms, *F(3, 726) = 0.31, p = .82, η^2 ^= .004*, diabetes treatment, *F(3, 726) = 0.82, p = .48, η^2 ^= .010* and diabetes complication, *F(3, 726) = 0.16, p = .92, η^2 ^= .002*.

*Bsc. Business Administration –* No significant difference was found for the general knowledge and awareness of diabetes, *F(3, 726) = 1.73, p = .16, η^2 ^= .022* and specific measures of diabetes symptoms, *F(3, 726) = 0.28, p = .82, η^2 ^= .004*, and diabetes treatment, *F(3, 726) = 0.55, p = .65, η*^2^ *^^= .007*. Only diabetes complication showed a significant difference among the levels of study, *F(3, 726) = 3.98, p = .01, η^2 ^= .49*. Multiple comparison with Bonferroni correction showed difference between level 100 and level 300 student, *p = .007*, with the latter having more knowledge and awareness about diabetes treatment with a mean(SD) of *7.00* (*2.91*) than the former, with a mean(SD) of *4.83* (*3.12*).

## Discussion

### Key results

Level of knowledge and awareness of type 2 diabetes was assessed in specific aspects of diabetes, namely, diabetes symptoms, treatment and complications. These specific aspects together formed participants’ general knowledge of diabetes. Overall, findings showed that although on average, about half of the participants made correct responses, there was poor knowledge and awareness of the specific aspects of diabetes measures. Participants had more knowledge and awareness of diabetes treatment than diabetes symptoms and complications. This is consistent with studies that have reported lack of knowledge and awareness of diabetes in for instance, Omani adults (Al Shafaee et al., 2008) and Jordanians students (Al-Sarayra & Khalidi, 2012), but contrary to findings of above average knowledge of diabetes, in a student population in Iraq (Mohammed et al., 2018) and among civil servants in Nigeria (Agu et al., 2014).

A possible explanation for this finding could be that participants made intelligent guesses when they responded to the questions, as several of the questions focused on diet and exercise, which are obvious guidelines for most health issues/clinical illnesses. This intelligent guess is made evident by the lower number of correct responses for more specific diabetes treatment questions, which focused on insulin injections, blood testing, foot problems, and hypoglycaemia. Thus, although participants had more knowledge and awareness of diabetes treatment, this may have been as a result of about half of the questions focusing on diet and exercise. Overall, questions that focused on dieting had the most correct responses, followed by questions on wounds and cuts, medication, weight management and controlled sugar levels. Questions, which had the lowest scores, were specific questions, which a person may only have knowledge of if he/she has a relative or a close associate with diabetes; or they were questions that were unrelated to diabetes.

This lack of knowledge in diabetes symptoms is consistent with findings reported by Al Shafaee et al. (2008) that most participants in their study lacked knowledge about basic symptoms of diabetes. Contrary to reports of low knowledge of diabetes management in a Pakistan sample (Gillani et al., 2018), in the present study, more than two-thirds of the participants had more knowledge and awareness of diabetes treatment. Overall, less than half of the participants from each of the disciplines of study had knowledge in the specific aspects of diabetes. More Family and Consumer Science students and Psychology students made correct responses for all the specific aspects of diabetes than Business Administration students did. On the other hand, Psychology students had more knowledge and awareness of diabetes symptoms than Family and Consumer Science students did, while the latter were more knowledgeable about diabetes treatment and complications.

Demographic variables, such as sex, degree of study, family history of diabetes, and lifestyles variables such as maintaining healthy eating and engaging in physical exercise, determined students’ knowledge and awareness of type 2 diabetes. Females had more knowledge and awareness of diabetes treatment than males, but they did not differ in their knowledge and awareness of diabetes symptoms and complications. This is consistent with findings of dos Santos, dos Santos, Ferrari, Fonseca, and Ferrari (2014) who reported females having more knowledge and awareness of diabetes than males. However it is contrary to findings that males were more knowledgeable about diabetes than females (Deepa et al., 2014; Fezeu et al., 2010; Gillani et al., 2018), and reports of no difference between Nigerian males and females (Agu et al., 2014). Perhaps, females reported more knowledge in diabetes treatment because about half of the questions focused on diet and exercise, and females have been reported to be more conscious about diet and weight loss issues (e.g. Brennan, Lalonde, & Bain, 2010; Thackray, Deighton, King, & Stensel, 2016).

Discipline of study also produced differences in knowledge and awareness of type 2 diabetes. Family and Consumer Science and Psychology students had more knowledge and awareness in both the general and specific aspects of knowledge and awareness of diabetes than Business Administration students did. Perhaps, students enrolled in the Family and Consumer Science and Psychology programmes may have studied human behaviour and may have learned about illnesses, weight management, or healthy lifestyles, compared to Business Administration students whose programme focuses generally on corporate and management issues, and not health issues. However, in comparing knowledge and awareness amongst the levels of study (100, 200, 300, 400) within each of the three disciplines, none of the comparisons showed significant differences in the level of knowledge and awareness of type 2 diabetes, except for a difference between level 100 and level 300 Business Administration students. It is not clear why this difference existed because if years of study affected the level of knowledge and awareness, then one would have expected significant differences between any of the lower and higher levels of study (for each discipline), especially levels 100 and 400, as reported by Al-Sarayra and Khalidi (2012). In addition, one would have expected that students offering more related courses (Family and Consumer Science and Psychology) would have shown difference among the levels of study but this was not the case. Overall, the findings suggest that how long a student had studied in a particular discipline did not affect their level of knowledge and awareness of type 2 diabetes.

Family history had an influence on students’ knowledge and awareness of type 2 diabetes. Students who had family history of diabetes had more knowledge and awareness of diabetes symptoms and treatment than those without family history of diabetes. This may be because the former probably live with or interact with family members who have been diagnosed and are on treatment. They are therefore likely to learn from their family members, which may increase their level of knowledge and awareness of type 2 diabetes. Sometimes also, those diagnosed with diabetes may caution family members (without diabetes) about their predisposition to the illness. This could make the latter more cautious and willing to learn about the illness, knowing that they may be vulnerable to the illness. This finding is consistent with the findings of Al-Sarayra et al., (2012) who reported the influence of family history on the knowledge and perception of diabetes, with two-thirds of the Jordanian students having good knowledge about diabetes. However, family history of diabetes amongst a Nigeria sample has been unrelated to their knowledge of diabetes (Agu et al., 2014).

Students who reported maintaining healthy eating had more general knowledge and awareness of type 2 diabetes than those who did not maintain healthy eating. This could be as a result of their better knowledge of diabetes complications, but not diabetes symptoms and treatment. A probable explanation is that students who reported healthy eating may be aware of the consequences/illnesses associated with poor eating, but not about the symptoms and treatment of illnesses, such as diabetes. The findings also showed better general knowledge and awareness of type 2 diabetes for students who engaged in physical exercise than those who did not, though this difference did not reflect in the specific measures of diabetes knowledge and awareness. Again, this difference in general knowledge and awareness may have resulted from students’ high knowledge in diet and exercise as indicated above.

Thus, overall, although students had some knowledge and awareness of diabetes, this was not adequate and thus they would require some education. Their knowledge in diet and exercise should, therefore, be linked to diabetes. Gatimu, Milimo, and Sebastian (2016) on assessing the prevalence rates and determinants of diabetes among older adults in Ghana recommended the need for diabetes prevention programme that will target young people in the hope to reduce the burden of diabetes at an older age. With the increase in the prevalence of diabetes worldwide, education and preventive programmes will go a long way to reduce prevalence, as over time, these younger adults become older adult. Participant reported that they obtained information mostly from newspapers and magazines and then from lectures and seminars, thus, these sources of information should be targeted during education.

### Limitations

There are some limitations to this study. First, students from only three Departments of the University of Ghana were studied, thus, the findings cannot be generalised to the entire population of University students. However, findings offer some information about how a student’s discipline of study can influence his/her knowledge and awareness of diabetes. Second, although during data collection, participants were informed that the questionnaire assessed type 2 diabetes, this was not specified in the items on the questionnaire. This was to avoid the repetitive use of the words ‘type 2 diabetes’. Perhaps the overall instructions for the scale could have indicated it was assessing knowledge in type 2 diabetes. Future studies using this scale should indicate in the instructions that it assesses knowledge and awareness of type 2 diabetes. Third, the Knowledge and Awareness of Diabetes Scale used is a new measure developed by the author (based on prior scales presented in the literature), and internal consistency for the overall scale and the subscales was good. However, as cautioned by Dima (2018), Cronbach’s alpha (and even exploratory factor analysis) is not always the best for psychometric analysis and thus, results may be misleading. Dima (2018) recommends a ‘6-step protocol’ for a meaningful psychometric analysis (e.g. structural validity, internal consistency), for scales that have binary and ordinal response choices measuring differences in degree or quantity (see Dima, 2018). Future studies using this scale should consider using this 6-step protocol for a meaningful psychometric analysis. Fourth, the lengthy nature of the questionnaires as mentioned by some participants may have caused them to complete the questionnaires in haste. Some participants even withdrew from the study as a result. Future studies can consider reducing the items on the scale. Finally, the inadequate self-report of participants’ weight and height did not make it possible for their BMI to be calculated and reported. Thus, future studies should consider measuring participants’ weight and height as part of the data collection process.

### Interpretation

Despite the increase in the prevalence of diabetes, knowledge and awareness of type 2 diabetes continues to be poor. A significant number of students lacked knowledge and awareness of type 2 diabetes, especially on specific questions related to the illness. Students were knowledgeable in exercise and dietary management issues but not in symptoms and complications of diabetes. The fact that the discipline of study produced differences in type 2 diabetes knowledge and awareness, suggests how education can make a difference in creating knowledge and awareness. Thus, education in diabetes is needed in order to make individuals more aware of the illness and take preventive measures. Unless individuals are educated about the symptoms and risk factors, the prevention of diabetes will be very much unlikely or will be an illusion. If individuals have little knowledge about the symptoms of diabetes more people with high sugar levels may go undiagnosed. They may be diagnosed opportunistically (by accident) and their condition may have become worse at that point, making them more susceptible to diabetes complications.

### Generalisability

Considering that participants were conveniently sampled for the study and not randomly sampled, finding cannot be generalised to all students in the three disciplines of study or to the entire population of the University of Ghana.
